# Effect of Spirulina (*Arthrospira platensis)* Supplementation on Physical and Chemical Properties of Semolina (*Triticum durum*) Based Fresh Pasta

**DOI:** 10.3390/molecules27020355

**Published:** 2022-01-06

**Authors:** Marianna Raczyk, Katarzyna Polanowska, Bartosz Kruszewski, Anna Grygier, Dorota Michałowska

**Affiliations:** 1 Department of Food Technology and Assessment, Institute of Food Sciences, Warsaw University of Life Sciences—SGGW, Nowoursynowska 159 C, 02-776 Warsaw, Poland; 2 Department of Food Technology of Plant Origin, Faculty of Food Science and Nutrition, Poznan University of Life Sciences, Wojska Polskiego 31, 60-637 Poznan, Poland; katarzyna.polanowska@up.poznan.pl (K.P.); anna.grygier@up.poznan.pl (A.G.); 3 Prof. Waclaw Dabrowski Institute of Agricultural and Food Biotechnology—State Research Institute, Rakowiecka 36, 02-532 Warsaw, Poland; dorota.michalowska@ibprs.pl

**Keywords:** spirulina, semolina, dietary fiber, protein, fatty acids, amino acids, color, sensory evaluation, pasta

## Abstract

Taking into account that many advantages have been associated with the consumption of spirulina (microalgae) in terms of antioxidant capacity, anticancer, anti-inflammatory, and anti-aging activities, the study focuses on spirulina supplementation of semolina-based pasta. Fresh pasta was prepared by mixing semolina flour (*Triticum durum*) with an addition of 3, 5, 7, and 10% (*w*/*w*) of spirulina *(Arthrospira platensis)* powder. Physicochemical and nutritional analyses were done on raw materials, and on fresh pasta before and after cooking. Sensorial analysis was done shortly after cooking pastas. Spirulina had a high content of protein (71.34%), with all the essential amino acids, a high total fiber (8.45%), as well as ash content (5.93%), which significantly increased the nutritional value of the obtained fresh pasta. Supplemented pastas have a significantly better amino acid profile and higher total fiber content (up to 2.99 g/100 g d.m.) than the control sample. Moreover, the addition of spirulina had a significant effect on the pasta’s color, weight gain, and cooking loss after being cooked. The addition of spirulina also affected the scores obtained for the individual parameters (texture, color, flavor, taste, and overall acceptability) of the sensory evaluation.

## 1. Introduction

Pasta, traditionally produced with durum wheat semolina, is a widely consumed food due to its palatability, low cost, and ease of production. Although the dry pasta market represents most of the world’s pasta consumption, the fresh pasta market share is continuously growing, associated with the consumer’s subconscious belief in the close relationship between freshness and artisanal production [1]. Fresh pasta is a perfectly suitable product for food fortification (e.g., fiber, vegetables, pulses, and microalgae), and it has received much attention from the industry and scientific communities [2,3].

Durum wheat is the second most cultivated species of wheat after *Triticum aestivum*. Semolina (*Triticum durum*) is used to produce high quality pasta, and it is coarser and has a darker (more golden) color compared to other wheat flours because of its higher carotenoids content [4]. Durum wheat also has more protein (av. 12% of protein) and a better gluten quality than common wheat (*T. aestivum*) (av. 9% of protein) [5]. Durum wheat grains can be ground into semolina to be used in pasta or couscous production; the exact size of the particles depends on type, origin, and wheat processing [6].

Currently, there is a big interest in increasing nutritional value in terms of the protein and fiber content of pasta [7]. Over the years, there have been many studies and articles focused on different protein materials such as beans, peas [8], shrimp meat [9], beef meat [10], fish powder [11,12], whey and milk powder [13]. However, taking into account more sustainable and high protein materials, pasta has a high potential to be enriched in spirulina (*Arthrospira platensis*), due to its simple manufacturing process, low cost, nutritional value, and high acceptance, including the green color [14]. Even though, in recent years, mass production of spirulina has increased rapidly, there is still a problem with keeping a stable and high quality in outdoor grown microalgae [14,15]. Thus, it is important to constantly maintain production standards and evaluate the quality of this product.

In reference to the current trends, this study aims at adding plant-based raw materials in order to improve the quality and nutritional value of fresh pasta. Spirulina seems to be a very good choice to increase the nutritional value and overall quality of fresh pasta because it has a high protein content (usually between 60–70% DM), high digestibility due to mucopolysaccharide cell wall, a low amount of nucleic acids, and a valuable amino acid profile, which is similar to the one recommended by FAO [16]. What is more, spirulina presents considerable proportions of other bioactive compounds such as carotenoids, chlorophylls, phycocyanin, vitamins (B1, B2, B3, B12), minerals (iron, copper, magnesium) and polyunsaturated fatty acids, including gamma-linolenic and linoleic acids [14,15,17]. Additionally, it has a green-blue color that is mostly positively perceived by consumers in this kind of product, as pasta. Furthermore, it is a sustainable source of macro- and micronutrients, and it can be consumed by vegan or vegetarian consumers. Spirulina is also sold as a supplement in the form of tablets, but implementing it into food products could be more attractive and convenient for customers [17]. In addition to human nutrition, spirulina is also used in animal and fish feeding, and some chemical compounds are extracted to produce pharmaceuticals and nutraceuticals.

The aim of the study was to investigate the nutritional and technological effects of *A. platensis* on semolina-based fresh pasta. The sensory quality of the product was also investigated, as spirulina supplementation can significantly alter its taste, color, texture, and odor. Thus, it is important to evaluate whether it will still be acceptable to potential consumers.

## 2. Results and Discussion

### 2.1. Characteristics of the Semolina Flour and Spirulina Powder

The characteristics of the raw materials used as main pasta ingredients are presented in Table 1. The moisture content of flour should be kept below 15% to be stable at room temperature. Undesirable microorganisms could start growing at a moisture level above 15%, leading to off flavors [18,19]. Raw materials used in this study were below the limit of semolina (12.91%) and spirulina (7.09%). Such a low moisture content (5 to 6%) of spirulina powder has also been reported in other studies [15,20].

The ash (minerals) content of wheat flour generally varies from 0.4 to 2.0% depending on the wheat variety and milling extraction. Semolina used in pasta preparation had an ash content of 1.1%, so it was within the range typical for wheat flour. However, spirulina is a plant-based material which is rich in minerals and vitamins, so as expected, the ash content was significantly higher (5.9%) than in semolina. Other studies also prove the high content of ash in spirulina powder (from 7.1 to 7.7%) [15,17,20].

The semolina used in this study has a protein content of 11.88%, which is in line with the results of other research [1,6]. Durum wheat has higher protein content compared to other varieties of wheat [5]. Spirulina powder has a very high concentration of proteins (71.34%), which makes it one of the richest sources of protein among plant-based materials. In other studies, the protein content was also significantly high, usually between 60–70% DM [2,3,20].

Wheat grains are low in fat. In this study, the 1.3% content of this macronutrient was evaluated. In other studies, higher values of 2.5–4.4% were obtained [4,6]. The tested spirulina powder had a very low fat content (0.4%), which was significantly lower than other spirulina powders reported in the literature (which typically had a fat content of 7.0%). This difference is due to the method of extraction and preparation (i.a. purification) of the spirulina extract for sale, both dependent on the manufacturer. The low fat content of both raw materials contributed to their relatively low energy (spirulina: 333.4 kcal/100 g DM; semolina: 341.3 kcal/100 g DM) (Table 1). However, based on the reviewed scientific articles, fat content of spirulina varies from 1.0 up to 12.0%, and largely depends on the growing conditions of the microalgae.

Semolina is mostly a source of carbohydrates, with a content of around 70% [4,6], which is in line with the obtained result (68.39%), and it mostly contains starch. The carbohydrates content of spirulina is rarely evaluated, but it should range around 5–20% [3,20]. The investigated spirulina powder had a low content (6.83%) of carbohydrates (Table 1).

### 2.2. Dietary Fiber Evaluation

According to the results of this study (Table 1), the total dietary fiber of spirulina (8.45%) was significantly higher than that of semolina (4.44%). Spirulina powders evaluated by other authors had a diverse fiber content of 3% [15], 7.93% [20], and 14.98% [21]. In other studies, the insoluble dietary fiber content was higher than the soluble fraction. It was also observed in our study (Table 1). Because of the many nutritional benefits of dietary fiber consumption, like lowering cholesterol levels, helping to control sugar levels, and maintaining bowel health, it is beneficial to enrich our diet with fiber [22,23].

**Figure 1:**
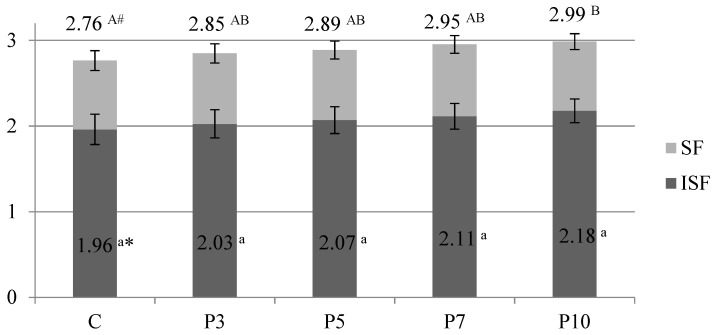
Fiber content in the analyzed pasta samples (g/100 g dry matter). C—control (semolina pasta); P3, P5, P7, P10—pasta samples with 3, 5, 7, 10 percentage replacement of semolina flour with spirulina powder. The bars show the total fiber content divided into a dark grey part (insoluble fiber content, ISF) and a light grey part (soluble fiber content, SF). Bars represent means ± standard deviation, (*n* = 3, for each pasta variant). * The values refer to the ISF content, marked with the same lowercase letter (a) are not significantly different (*p* < 0.05). # The values refer to the total fiber content, marked with different capital letters (A, B) are significantly different (*p* < 0.05).

**Figure 2:**
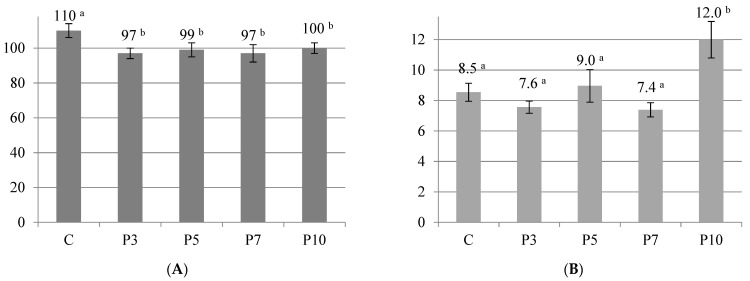
(**A**) Weight gain [%] and (**B**) cooking losses [%] obtained for pasta samples. C—control (semolina pasta); P3, P5, P7, P10—pasta samples with 3, 5, 7, 10 percentage replacement of semolina flour with spirulina powder. The values represent means ± standard deviation (*n* = 3). The values in a row with different letters (a, b) are significantly different (*p* < 0.05).

The obtained pastas contained similar total fiber contents (2.76–2.99 g/100 g) DM after cooking (Figure 1). In this respect, only the pasta with the highest content of spirulina P10 differed statistically significantly from the control sample. Pasta enriched with 10% spirulina powder can be considered a good source of total dietary fiber. As the spirulina share increased, the content of insoluble fiber also increased slightly in the pasta samples. The content of soluble fiber in pasta remained almost unchanged between 0.80 and 0.84 g/100 g DM. The incorporation of spirulina powder into semolina led to a lower absorption of water by the pasta (Figure 2A). Moreover, the cooking loss increased due to the dilution of the protein network by fiber. The escalation of this phenomenon is well illustrated in pasta with 10% spirulina (Figure 2B). A similar phenomenon was observed when pasta was enriched with dried and powdered fruits and vegetables [24].

### 2.3. Physicochemical Characteristics and Cooking Properties of Pasta

The optimal cooking time was the same for all variants of pasta (five min). The initial water content of fresh pasta was already high (36%). Thus, the weight gain (water absorption) was relatively low, in a range between 97 and 110%, depending on the pasta variant, and it was the highest in the control sample (100% semolina pasta) (Figure 2A). Pastas with 3 and 7% of spirulina (P3 and P7) had the lowest weight gain, but also the lowest cooking loss (Figure 2B).

Pasta with 10% of spirulina (P10) had the highest cooking loss (12%), which could be an effect of protein solubilization and washing out of the protein fraction and a weakening of the gluten matrix at the same time. A weaker gluten matrix is less able to entrap swollen starch granules during boiling [3,20].

The enrichment with spirulina powder significantly affected the color of the pasta dough, as evidenced by the high values of ΔE* (Table 2). When compared to the control sample, spirulina supplementation significantly darkened the pasta dough (L* parameter). However, there is no difference in the L* parameter between samples P7 and P10. As expected, the supplementation of spirulina resulted in the greener color of the pasta. However, the greatest difference in parameter a* was between the control and the 3% spirulina samples. The addition of spirulina also caused a significant decrease in the b* color parameter. It was found that there were no significant differences between samples P5, P7, and P10. Nevertheless, it should be noted that for each color parameter there was a limit of 7% spirulina addition, above which no statistical difference was found. A sensory evaluation of the P7 sample achieved the highest scores, also in color evaluation. When pasta or bread are enriched with some plant or animal materials, various color changes are obtained [12,13,25,26]. According to the literature, these changes are not always acceptable to the consumer and may be rejected despite the increased nutritional value associated with the health benefits [24]. But in our experiment, it was possible to meet the increased nutritional value of pasta with good consumer acceptance. However, this solution may not work in other countries or cultures because of different preferences.

### 2.4. Amino Acids and Fatty Acids Evaluation

Spirulina mostly contains proteins with amino acids, as shown in Table 3. *Arthrospira platensis* provides a complete set of eighteen amino acids including all the exogenous ones [3,21]. It has a relatively high content of lysine (22.62 mg/g), an amino acid that is lacking in wheat flour. It is also a good source of other essential amino acids such as histidine, threonine, phenylalanine + tyrosine, valine, leucine, isoleucine, and methionine + cysteine (Table 3). Glutenin and gliadins are recognized as major wheat storage proteins that have an important role in the determination of pasta and other cereal products’ quality. However, lysine and methionine are not at a satisfactory level in durum wheat. When combined with spirulina, it becomes an excellent nutritional material. The content of lysine and methionine, as well as other amino acids, significantly increased in the fresh pastas (Table 4). From the nutritional point of view, supplementation with 10% of spirulina powder is the best in terms of protein content. The amino acid profiles of the evaluated semolina and spirulina (Table 3) are in line with the results of other authors [27,28]. Proteins are essential nutrients for the proper functioning of the body, as they are its main building components.

Semolina and spirulina have different compositions of fatty acids. Durum wheat mostly consists of polyunsaturated fatty acids (linoleic acid: 56.02% and alpha-linolenic acids: 3.45%), followed by monounsaturated fatty acids (dominated by oleic acid: 20.79%) and the lower content of saturated fatty acids (mostly palmitic acid: 16.83%) (Table 3). Those results are compatible with the ones reported by other authors [29,30]. On the other hand, spirulina was found to have the highest content of saturated fatty acids (56.63%), mostly palmitic acid (51.54%), followed by PUFAs, the main shares of which were gamma-linolenic (19.30%) and linoleic acids (18.51%), and a low amount of MUFAs (5.57%), with the main contributions of palmitoleic (2.88%) and oleic (2.69%) acid. According to other authors, spirulina is characterized by a higher content of PUFAs (40–50%) [4,6,21], than what was observed in our study. Based on the results of other authors, spirulina has a high content of gamma-linolenic acid and a significant content of alpha-linolenic acid (around 10–15%). However, as with the fat content of spirulina, the fatty acid composition also varies greatly depending on growing conditions [4,6,21].

### 2.5. Sensory Characteristics of Pastas

**Figure 3:**
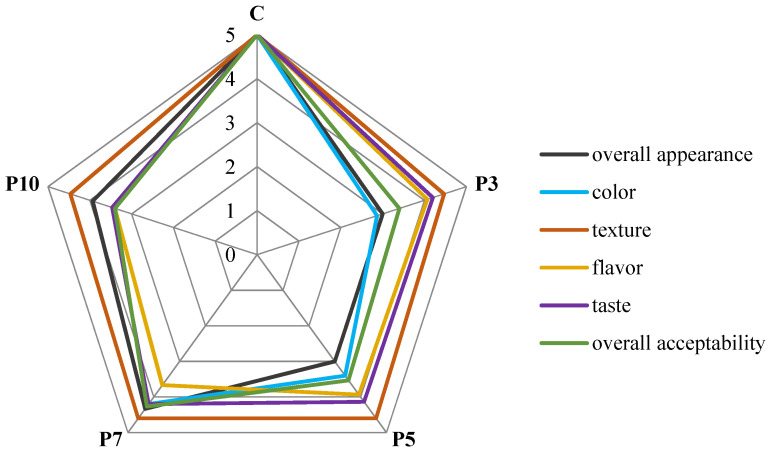
The scores of the sensory analysis of the obtained fresh pastas. C—control (semolina pasta); P3, P5, P7, P10—pasta samples with 3, 5, 7, 10 percentage replacement of semolina flour with spirulina powder.

From the average scores (Figure 3) of the sensory parameters evaluated in the study, it is observed that the control sample was preferred by the panel. Among the supplemented pastas, P7 (with 7% of spirulina) obtained the highest notes for overall appearance, color, taste, and texture. Regarding the fresh pasta flavor, the consumers gave lower scores for pasta with the higher addition of spirulina powder. Interestingly, the pasta with an addition of 3% spirulina got the lowest scores for color, texture, and overall appearance. In another study [21] the organoleptic acceptability of noodles and macaroni got the highest scores for products supplemented with 2 and 4% spirulina powder, but a sensory evaluation had not been done. Other studs have found a favorable sensory evaluation of gluten-free pasta supplemented with spirulina compared to wheat and rice pastas [3].

### 2.6. The Comprehensive Overview of Obtained Pastas Characteristics

**Figure 4:**
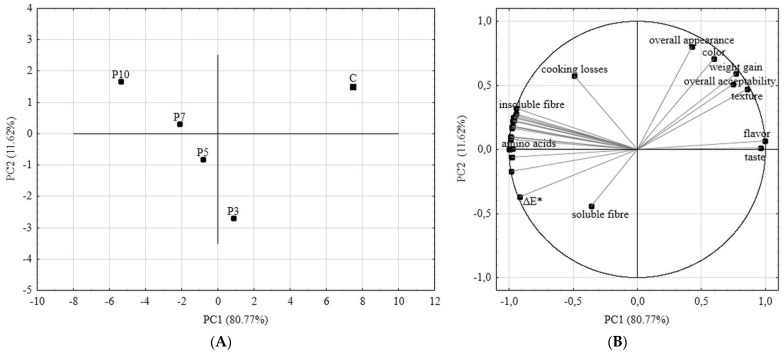
PCA analyses results. (**A**) Score plot, PC1 versus PC2 of all samples; (**B**) Score plot, PC1 versus PC2 of data from determinations used as variables. C—control (semolina pasta); P3, P5, P7, P10—pasta samples with 3, 5, 7, 10 percentage replacement of semolina flour with spirulina powder.

In order to demonstrate the alterations occurring in the types of the obtained pastas, the chemometric analysis in the form of the principal component analysis (PCA) was conducted on the qualified gathered data. In the PCA analysis, the four principal components were created, of which the first two explained 92.39% of the total variability. Figure 4a shows the distribution of samples in two-dimensional space relative to the first (PC1) and second (PC2) principal components. The control sample was well separated from the rest of the pasta samples as a result of distinctive physicochemical and sensory characteristics. Also, the samples of pasta with spirulina are spaced far apart, which indicates that each time the flour content is partially replaced by another 2–3% of spirulina, the product characteristics change significantly. This should be taken into account in future formulation trials using spirulina.

The attributes that most differentiated the samples of pastas with spirulina powder were the content of insoluble fiber, individual amino acids, total color difference ΔE*, and value from flavor evaluation (Figure 4B). These attributes were strictly influenced by the amount of spirulina powder added in made pastas. Insoluble fiber and individual amino acid content were increasing with the increase of spirulina in the product, because as has been shown in the characteristics of raw materials used for this study, spirulina is very abundant in proteins and abundant in insoluble fiber. Due to its richness in distinctive natural pigments including chlorophylls, carotenoids and C-phycocyanin [31], spirulina has a strong influence on the color of the product, which is associated with obtained total color difference values. Commercially sold spirulina powder has a specific flavor that was reflected in the tested samples.

## 3. Materials and Methods

### 3.1. Chemicals

All the reagents and solvents used were of the reagent grade purity, they were obtained from Avantor Performance Materials Poland S.A. (Gliwice, Poland).

### 3.2. Preparation of Pastas

The pastas were made according to recipes given in Table 5. The doughs were obtained by mixing and kneading semolina (country of origin: Poland), water (36%) and spirulina powder (country of origin: China) in a proportion to semolina (3, 5, 7 and 10%). The dough was kneaded for 20 min by hand and rolled. Once each dough was evenly mixed, it was rolled to a thickness of 2 mm and cut into tagliatelle shapes (long ribbons). The dough was rolled and cut using a Marcato Atlas 150 machine (Italy). The pasta was dried for half an hour at room temperature and cooked (100 g of pasta in 1 L of tap water with 7 g of salt). Cooking time was determined as 5 min.

### 3.3. Weight Gain and Swelling Index after Cooking

The weight gain of pasta was determined according to that described by Cleary and Brennan (2006) [32], with the following modifications: 100 g of pasta was cooked in 1 L of water during the optimal cooking time (5 min) and was cooled in 2 L of cold water; then the pasta was dried with absorbent paper and weighed in an analytical balance. To obtain the percentage of the total weight gain of cooked pasta, the following equation was applied:

$$\mathrm{Weight}\;\mathrm{gain}=\frac{\mathrm{cooked}\;\mathrm{pasta}\;\mathrm{weight}-\mathrm{raw}\;\mathrm{pasta}\;\mathrm{weight}}{\mathrm{raw}\;\mathrm{pasta}\;\mathrm{weight}}\;\times \;100$$

### 3.4. Cooking Losses

The AACC 66-50 method [33] was carried out with the same characteristics as in the previous section (100 g of pasta in 1 L of water, cooked for 5 min). The water resulting from the cooking was collected in crucibles and evaporated at 105 °C until reaching a constant weight (24 h). The dry residue was weighed on an analytical balance and determined as a percentage of the total weight of the pasta before cooking.

### 3.5. Moisture and Ash Determination

The flour and pasta moisture was determined by the drying method according to the AACC standard method 44-15.02 [34]. 5 g of each sample was weighed on an analytical balance into metal dishes, and then dried in a SUP-65 W laboratory dryer (Wamed, Poland) at 105 °C for 24 h. After drying, the dishes were placed in a desiccator to cool and then reweighed. The moisture was calculated from the mass difference. The ash content was determined based on the AACC standard method 08-01.01 [35]. 5 g of each flour was weighted into an ash dish. Then samples were placed in a muffle furnace at 900 °C for 1 h. After cooling, the samples were weighed, and the ash contents were calculated.

### 3.6. Determination of Proximal Nutritional and Energy Values

Protein content was determined according to the Kjeldhal method [36], with correction factors of 5.7 (wheat) and 5.95 (spirulina) as other authors reported [37,38], whereas fat was extracted and determined by Soxhlet apparatus using diethyl ether as solvent. Total dietary fiber content, including insoluble and soluble fractions, was determinate by the enzymatic-gravimetric method according to AOAC 991.43, and AACC 32-07 procedures [39,40]. For these determinations, the Fibertec^TM^ E System (FOSS, Hilleroed, Denmark) was used. The carbohydrates content was calculated by subtracting the values of the protein, fat, moisture, total dietary fiber and ash content from 100. The energy values (kcal) were calculated using conversion factors according to EU Regulation No 1169/2011 (4 kcal per g for protein and carbohydrates, 9 kcal per g for fat, 2 kcal per g for total fiber). The conversion factor for kilojoules is 1 kcal = 4.184 kJ.

### 3.7. Amino Acids Determination

The amino acid profile of the samples was determined by an HPLC gradient system with precolumn phenylisothiocyanate (PITC) derivatization after acid hydrolysis in 6 M HCl with 1% phenol under nitrogen at 110 °C during 24 h as proposed by Kwanyuen and Burton (2010) [41]. For gradient two buffers were used: buffer A (0.1 M ammonium acetate, pH 5.14) and buffer B (0.1 M ammonium acetate containing acetonitrile 40:60, *v*/*v*). The sample was prepared as follows: 100 μL of sample was dried under vacuum, then 80 μL of coupling reagent was added (methanol, water, triethylamine [TEA] [2:2:1, *v*/*v*/*v*]), mixed well and dried under vacuum. Subsequently 80 μL of PITC reagent (methanol/PITC/TEA/water [7:1:1:1; *v*/*v*/*v*/*v*]) was added and kept at room temperature for 30 min for reaction. Then the PITC was removed under vacuum and the derivatized sample was redissolved in 1.5 mL of mixture of buffer A and methanol 50:50 *v*/*v*. The sample was filtered through syringe filter (0.45 μm) and 10 μm injected to the HPLC system. The temperature of the column was kept at 39 °C. The measurements were taken at the absorbance of 254 nm. The identification was based on retention times of analytical standards. Norleucine was used as an internal standard. The analysis was performed using LC Agilent Technologies 1200 Rapid Resolution (Santa Clara, CA, USA) system equipped with a UV-Vis detector DAD 1260 (Santa Clara, CA, USA) and a reversed-phase column Zorbax Eclipse Plus C18 (4.6 × 150 mm, 5 µm) (Santa Clara, CA, USA). All HPLC analyses were performed in duplicates.

### 3.8. Color Determination of Pastas Dough

The color of fresh pasta dough slices before cutting and before cooking was evaluated using a colorimeter Konica Minolta CM-3600d (Tokyo, Japan), calibrated against black and white plate standards. Dough color was determined at the middle point of the central 2 mm thick slice. The parameters of the device in the reflection mode were set as follows: a standard observer of 10°, and an illuminate D65. The measurements were performed at room temperature through a diaphragm of 3 cm in diameter. The color was expressed in the CIE L*a*b* scale, and the parameters determined were: L* (lightness), a* (redness) and b* (yellowness).

### 3.9. Fatty Acid Composition

Fatty acid composition was determined using a previously reported method [42]. The fat was extracted from samples using hexane (>99%, Avantor, Gliwice, Poland). The hexane-flooded samples were shaken for 1 h. Then 1 mL of hexane was used for fatty acid methylation. 1 mL of 0.4 M sodium methoxide (Merck, Darmstadt, Germany) was added to the fat dissolved in hexane and allowed to stand for 15 min. After that, 5 mL of distilled water was added and the upper hexane layer was taken for analysis (1 µL). The gas chromatograph was a Trace 1300 with FID detector (Thermo Scientific, Waltham, MA, USA), while a SP TM-2560 capillary column (100 m × 0.25 mm × 0.2 µm) (Supelco, Bellefonte, PA, USA) was used for the analysis. The carrier gas was hydrogen (1.5 mL/min). The analysis was performed in splitless mode. The initial oven temperature was 160 °C for 1 min, and then it was increased 6 °C/min to 220 °C and it remained at this temperature for 17 min. The inlet and detector temperature were 240 °C. The retention times were compared to the retention times of 37 Component Fame Mix (Supelco, USA).

### 3.10. Sensory Evaluation of Fresh Pastas

Sensory evaluation of the pastas was done by ten trained panelists on a day of production (shortly after cooking). The samples were served without any additives at a temperature of around 60 °C following the recommendations of ISO 6658:2017 [43]. The questionnaire included a 10-point scaling method based on the Polish norm (PN-A-74108, 1996). The group of trained panelists received samples of coded pastas. During the testing sessions, panelists had access to drinking water to clean their palates between evaluations. Panelists were trained using ISO 8586-1:2012, ISO 8586-2:2014, and ISO 11036:2020. Pasta was first evaluated before cooking by the following characteristics: appearance (0 = irregular and 5 = regular), color (0 = uneven, discouraging 5 = alignment), visible contaminants content (present or absent). After cooking, the pastas were cooled down in cold water and evaluated by the following characteristics: appearance (0 = irregular and 5 = regular), color (0 = uneven, discouraging, 5 = alignment), firmness (0 = very soft or hard, 5 = al dente), taste (0 = bitter and 5 = pleasant, typical for wheat pasta), flavor (0 = foreign, 5 = typical, lack of foreign or unpleasant odor) and overall acceptability (0 = dislike very much and 10 = like very much).

### 3.11. Calculations and Statistics

All the data in the tables was presented as a mean with standard deviations. Unless otherwise stated, determinations were performed in triplicate. The statistical program Statistica 13.3 (TIBCO Software Inc., Palo Alto, CA, USA) was used to develop the results. The effect of the semolina flour replacement with spirulina powder on nutrition composition and physicochemical properties was analyzed using one-way analysis of variance (ANOVA). To evaluate the differences between average values for data that was normally distributed, the Tukey HSD test was used, with a significance level of α = 0.05. If the tested data did not come from normal distribution, the Kruskal–Wallis test was used instead.

The gathered quantitative data were used in the chemometric analysis in principal component analysis (PCA) in order to show the changes in characteristic of the obtained spirulina supplemented pastas versus the control sample. The results of the determinations performed were qualified for PCA analysis based on a correlation score with the first or second principal component at a level of at least 0.6 [44]. According to the generated factor loadings matrix, data from the following analyses of pastas were qualified for PCA classification: insoluble and soluble fiber, all the individual amino acids, weight gain, cooking losses, total color difference ΔE*, and sensory characteristics (overall appearance, color, texture, flavour, taste, overall acceptability).

## 4. Conclusions

This study concludes that the supplementation of semolina-based fresh pasta with spirulina has a positive effect on nutritional quality in terms of protein and amino acid content. Among other physicochemical characteristics, it strongly affected the color of the obtained product. Cooking properties were similar to those of the control sample, except for a 10% replacement of flour with spirulina powder. Based on the sensory evaluation, the sample with 7% of spirulina got the highest scores among all the analyzed supplemented fresh pastas. Obtaining a fortified pasta product with acceptable quality is often a challenge, and the proportion of individual additives should be precisely determined considering the preferences of consumers. In this study, the increased nutritional value of fresh pasta was achieved with good consumer acceptance. However, based on the results of sensory analysis, future research should be aimed at improving the sensory characteristics of pasta with the greater addition of spirulina powder (>10%) to provide higher nutritional value in alignment with consumer acceptance. Also, elemental composition studies of pasta with spirulina should be done to learn another health aspect of spirulina food enrichment.

## Figures and Tables

**Table 1:** Proximate macronutrients content (g/100 g of dry matter) and energy value of the spirulina powder and semolina flour.

	Spirulina Powder	Semolina Flour
Moisture (%)	7.09 ± 0.04 ^a^	12.91 ± 0.01 ^b^
Protein	71.34 ± 0.21 ^b^	11.88 ± 0.12 ^a^
Fat	0.36 ± 0.01 ^a^	1.26 ± 0.05 ^b^
Ash	5.93 ± 0.30 ^b^	1.12 ± 0.14 ^a^
Carbohydrates	6.83 ± 0.19 ^a^	68.39 ± 0.08 ^b^
Insoluble dietary fiber	6.46 ± 0.33 ^b^	3.07 ± 0.28 ^a^
Soluble dietary fiber	1.99 ± 0.14 ^a^	1.37 ± 0.20 ^a^
Total dietary fiber	8.45 ± 0.19 ^b^	4.44 ± 0.08 ^a^
Energy value (kJ)	1395.0 ± 0.9 ^a^	1427.9 ± 3.5 ^b^
Energy value (kcal)	333.4 ± 8.5 ^a^	341.3 ± 0.8 ^b^

The values represent means ± standard deviation (*n* = 3). The values in a row with different letters (a, b) are significantly different (*p* < 0.05).

**Table 2:** Color values in CIE L* a* b* system of the pasta doughs supplemented with spirulina powder.

Color Values	C	P3	P5	P7	P10
L*	75.26 ± 1.25 ^d^	40.18 ± 1.07 ^c^	35.22 ± 0.68 ^b^	32.56 ± 1.86 ^a^	32.01 ± 0.92 ^a^
a*	3.45 ± 1.01 ^d^	−14.12 ± 0.56 ^a^	−11.45 ± 0.18 ^b^	−9.88 ± 0.74 ^c^	−8.72 ± 0.39 ^c^
b*	27.83 ± 2.27 ^c^	7.21 ± 0.30 ^b^	5.90 ± 0.20 ^ab^	5.21 ± 0.45 ^a^	4.71 ± 0.25 ^a^
ΔE*	-	44.34 ± 0.76 ^a^	48.03 ± 0.56 ^b^	50.14 ± 1.58 ^c^	50.53 ± 0.80 ^c^

C—control (semolina pasta); P3, P5, P7, P10—pasta samples with 3, 5, 7, 10 percentage replacement of semolina flour with spirulina powder. The values represent means ± standard deviation (*n* = 5). The values in a row with different letters (a, b, c, d) are significantly different (*p* < 0.05).

**Table 3:** Amino acids [mg/g dry matter] and fatty acids [%] profile of the spirulina powder and semolina flour.

Amino Acids	Spirulina Powder	Semolina Flour	Fatty Acids	Spirulina Powder	Semolina Flour
aspartic acid + asparagine	30.69 ± 0.74 ^b^	7.79 ± 0.32 ^a^	C14:0	nd	0.06 ± 0.01
glutamic acid + glutamine	49.63 ± 0.16 ^b^	40.16 ± 0.45 ^a^	C15:0	nd	0.09 ± 0.03
serine	20.35 ± 0.04 ^b^	8.46 ± 0.47 ^a^	C16:0	51.54 ± 2.26 ^b^	16.83 ± 0.49 ^a^
glycine	21.26 ± 0.32 ^b^	5.48 ± 0.00 ^a^	C16:1	2.88 ± 1.57 ^b^	0.18 ± 0.05 ^a^
histidine	27.39 ± 0.25 ^b^	3.94 ± 0.14 ^a^	C17:0	4.04 ± 0.13 ^b^	0.07 ± 0.00 ^a^
arginine	26.13 ± 0.44 ^b^	6.65 ± 0.05 ^a^	C17:1	nd	0.26 ± 0.05
threonine	39.61 ± 0.13 ^b^	4.97 ± 0.00 ^a^	C18:0	1.06 ± 0.28 ^a^	1.25 ± 0.07 ^a^
alanine	12.10 ± 0.27 ^b^	2.45 ± 0.09 ^a^	C18:1	2.69 ± 0.26 ^a^	20.79 ± 0.40 ^b^
proline	22.92 ± 0.00 ^a^	31.90 ± 0.04 ^b^	C18:2t	1.30 ± 0.04	nd
tyrosine	20.34 ± 0.37 ^b^	7.16 ± 0.18 ^a^	C18:2c	17.21 ± 0.74 ^a^	56.02 ± 0.37 ^b^
valine	27.89 ± 0.52 ^b^	6.43 ± 0.23 ^a^	C18:3 (n-3)	nd	3.43 ± 0.10
methionine	8.07 ± 0.14 ^b^	2.01 ± 0.01 ^a^	C18:3 (n-6)	19.30 ± 0.59	nd
cysteine	4.60 ± 0.06 ^a^	5.49 ± 0.01 ^a^	others	-	1.05 ± 0.03
isoleucine	25.34 ± 0.40 ^b^	5.11 ± 0.38 ^a^	Σ SFA	56.63 ± 0.89 ^b^	18.46 ± 0.08 ^a^
leucine	39.69 ± 0.80 ^b^	8.49 ± 0.32 ^a^	Σ MUFA	5.57 ± 0.92 ^a^	22.06 ± 0.14 ^b^
phenylalanine	19.02 ± 0.72 ^b^	7.59 ± 0.33 ^a^	Σ PUFA	37.80 ± 0.76 ^a^	59.50 ± 0.16 ^b^
lysine	22.62 ± 0.01 ^b^	7.57 ± 0.42 ^a^			

Σ SFA—total saturated fatty acids; Σ MUFA—total monounsaturated fatty acids; Σ PUFA—total polyunsaturated fatty acids; nd—not detected. The values represent means ± standard deviation (*n* = 3). The values in a row for specific determination with different letters (a, b) are significantly different (*p* < 0.05).

**Table 4:** Amino acids [mg/g dry matter] profile of the pasta samples supplemented with spirulina powder.

Amino Acids	C	P3	P5	P7	P10
aspartic acid + asparagine	7.17 ± 0.04 ^a^	9.61 ± 0.02 ^b^	13.21 ± 0.15 ^c^	14.81 ± 0.27 ^d^	18.99 ± 0.14 ^e^
glutamic acid + glutamine	28.95 ± 0.06 ^a^	33.87 ± 0.22 ^b^	40.38 ± 0.59 ^c^	43.95 ± 0.40 ^d^	48.18 ± 0.75 ^e^
serine	4.21 ± 0.02 ^a^	6.03 ± 0.06 ^b^	6.16 ± 0.20 ^b^	6.95 ± 0.20 ^bc^	8.05 ± 0.00 ^d^
glycine	2.68 ± 0.16 ^a^	3.79 ± 0.21 ^b^	4.15 ± 0.22 ^bc^	4.70 ± 0.19 ^cd^	5.22 ± 0.03 ^d^
histidine	0.71 ± 0.01 ^a^	2.97 ± 0.16 ^b^	2.96 ± 0.09 ^b^	3.27 ± 0.07 ^b^	4.05 ± 0.30 ^c^
arginine	3.03 ± 0.07 ^a^	4.59 ± 0.20 ^b^	4.91 ± 0.08 ^bc^	5.28 ± 0.05 ^c^	6.04 ± 0.21 ^d^
threonine	2.72 ± 0.03 ^a^	3.93 ± 0.14 ^b^	4.35 ± 0.08 ^c^	5.08 ± 0.13 ^d^	6.29 ± 0.10 ^e^
alanine	1.24 ± 0.00 ^a^	2.21 ± 0.01 ^b^	3.05 ± 0.15 ^c^	3.41 ± 0.05 ^d^	4.06 ± 0.08 ^e^
proline	10.35 ± 0.02 ^a^	13.99 ± 0.62 ^b^	18.87 ± 0.00 ^c^	20.55 ± 0.67 ^c^	23.59 ± 0.54 ^d^
tyrosine	2.58 ± 0.16 ^a^	4.11 ± 0.46 ^b^	3.91 ± 0.18 ^b^	4.48 ± 0.04 ^bc^	5.10 ± 0.06 ^c^
valine	3.76 ± 0.01 ^a^	5.44 ± 0.07 ^b^	6.56 ± 0.46 ^c^	6.98 ± 0.04 ^c^	7.25 ± 0.09 ^c^
methionine	1.42 ± 0.14 ^a^	2.37 ± 0.04 ^b^	2.39 ± 0.05 ^b^	2.79 ± 0.01 ^c^	3.47 ± 0.14 ^d^
cysteine	3.17 ± 0.07 ^a^	3.56 ± 0.02 ^b^	4.28 ± 0.10 ^c^	4.33 ± 0.01 ^c^	4.90 ± 0.11 ^b^
isoleucine	3.01 ± 0.03 ^a^	4.55 ± 0.40 ^b^	5.76 ± 0.16 ^c^	6.59 ± 0.01 ^d^	7.51 ± 0.14 ^e^
leucine	5.71 ± 0.25 ^a^	7.55 ± 0.14 ^b^	11.00 ± 0.70 ^c^	12.36 ± 0.21 ^cd^	14.11 ± 0.59 ^d^
phenylalanine	4.12 ± 0.09 ^a^	5.69 ± 0.07 ^b^	6.24 ± 0.21 ^b^	7.48 ± 0.21 ^c^	8.34 ± 0.11 ^d^
lysine	1.58 ± 0.11 ^a^	4.04 ± 0.38 ^b^	4.94 ± 0.10 ^c^	5.08 ± 0.07 ^c^	6.54 ± 0.19 ^d^

C—control (semolina pasta); P3, P5, P7, P10—pasta samples with 3, 5, 7, 10 percentage replacement of semolina flour with spirulina powder. The values represent means ± standard deviation (*n* = 3). The values in a row with different letters (a, b, c, d, e) are significantly different (*p* < 0.05).

**Table 5:** Recipe ingredients of studied pastas [% content].

Ingredient	C	P3	P5	P7	P10
Semolina flour	64.0	62.1	60.8	59.5	57.6
Spirulina powder	0.0	1.9	3.2	4.5	6.4
Water	36.0	36.0	36.0	36.0	36.0

C—control (semolina pasta); P3, P5, P7, P10—pasta samples with 3, 5, 7, 10 percentage replacement of semolina flour with spirulina powder.

## Data Availability

All data created and analyzed during the experiments was presented in this study.

## Sample Availability

Samples of the compounds are not available from the authors.
